# Event and Comment

**Published:** 1909-10-15

**Authors:** 


### DENTAL REGISTER.

**Vol. LXIII. October 15, 1909. No. 10.**

### EVENT AND COMMENT.

THE MILLER DENTAL CLUB. One of the most delightful pleasures of the American visitors to the International Dental Congress, in Berlin, was the cordial welcome extended them by the American Dentists practicing in Germany. It was most generous on all occasions, but was particularly impressive on the occasion of the banquet given by the W. D. Miller Club, of Berlin, Wednesday evening, August 25th, and this banquet at the Hotel Kaiserhof, will not soon be forgotten by those who attended. We have attended a great many banquets, but we never saw a more fraternal gathering, a more beautifully conducted banquet nor a happier lot of men. There is always a feeling of greater sympathy when one meets a man of his own country in a distant land, but when you get more than a hundred native Yankees together around a most bountifully supplied table and under circumstances calculated to call out every one's patriotism, it is no wonder that every one felt that it was the happiest moment of his life. The whole affair was most delightfully planned, and carried out with such tact as made every one glad to have made the trip across the Ocean for this occasion. The best of it all was that the Americans who gave it seemed to enjoy it almost as much as the rest of us. There was at first a tone of sadness at the remembrance of that great and noblest soul of all who was not there to enjoy it with us; but there was a universal expression that his spirit was present to approve and inspire us to the nobler and higher conceptions of a calling for which he heroically sacrificed his life. It was not a frivolous occasion but one of delightful fellowship with a conscious sentiment of loyalty to a noble and dignified calling and to those great spirits who have lead and inspired us at all times. We wish we could transfer the spirit and effect of that meeting to this printed page, it must be experienced to be realized.

THE FIFTH INTERNATIONAL DENTAL CONGRESS. Another great International Congress has passed into history and the results perhaps can never be counted by any one. This was the largest international assemblage of dentists ever known. More than 2,200 dentists frompractically all nations met in Berlin at the call of the German Central Verein and under the direction of the International Federation Dentaire. The largest number of dentists came from Germany. The American representation was not what was expected; 78 dentists from the United States were registered. The American delegation had no organized representation. There were only two members of the National Committee present and none of the officers. The essayist appointed to represent the National Dental Association was ill and could not attend. No one was authorized to speak for our country; and with no preparation Dr. J. Y. Crawford kindly spoke a few words of greeting for his countrymen. The program was so great that it was practically impossible to present it orderly or completely. Nearly 400 papers, lectures, demonstrations and clinics were scheduled. To present all this in five days to over 2,000 people required great skill and diplomacy, so that all would feel satisfied. The truth is that there was much complaint, but when it was all through every one felt that the management had really accomplished a tremendous task and with great credit. There were twelve sections, and each had all the material it could crowd into the time at its disposal. Sometimes it was absolutely necessary to change the time scheduled for a paper, which of course disconcerted those who had made their plans to attend the particular section at the time announced for some particular subject in which they were interested, only to find that some other paper had the floor, all of which made confusion and much adverse criticism. There was however plenty of interesting material being presented at all times in every section, and one could scarcely feel that his time was not well employed wherever he happened to be if he knew the language. The meetings were held in the German Reichstag Building, which was ideal for such a meeting. The large hall for the general meetings, the committee rooms for the sections, and the corridor for the exhibits, supplied every need in the most satisfactory manner. The section rooms were badly ventilated and not therefore conducive to quickness of thought. The clinics were perhaps the least important part of the Congress and perhaps this is explained by the fact that there was no suitable place for giving them. One of the most interesting features of the Congress was the scientific exhibit. There probably was never before brought together a more interesting anatomical and pathological exhibit, and probably in no other country could such an exhibit be produced. The museums, public and private, of Europe, must have contributed to make this remarkable display. There were crania showing perfection in development, and monstrosities in degenerative conditions, histologic and pathologic studies in abundance, showing that the German dentist is wide awake to the necessity of researches in these fundamental sciences, and it was frequently remarked that American dentistry must look to its laurels or not many generations hence they will pass over the seas, because of the tremendous strides our German friends have made in the study of the fundamental sciences. There can be no question that we may for a time hold the lead technically, but we can't do even this and neglect, the way we are now doing, to establish a true basis upon which we must ever construct all our technical procedures. Our confreres across the water are not neglecting the technical side of the profession, as the commercial exhibit clearly showed. There is not a single instrument or appliance of consequence made or used in this country that is not made or to be had in Germany, and they have many helps to practice which in time will find their way to our country. It is true that the German made goods are in quality and design usually inferior. At present they seem disposed to copy our instruments and make them cheap. For instance, we saw beautifully wrought forceps that sold there for one dollar and fifteen cents that would cost of American or English make, three dollers. American dentists practicing in Germany told me that they did not find it economical to buy German instruments as compared with American or English manufacture. All this will surely change as time goes on and Germany will make good dental as well as surgical instruments. The social features of the Congress were all that any one could desire. The Imperial government, the City of Berlin and the profession of Germany did all that the most exacting could wish to make their guests comfortable and at home. Berlin is a new and modern built City; it is one of the best governed and the most sanitary cities in the world. There are no slums, there is no rowdyism. The people have the most wholesome respect for law and order, and life and property are sacred and protected by a most efficient police department. The grass in the parks is to look at and so are the flowers for everyone to enjoy, but no one ever thinks of taking liberties with them. The parks are filled with beautiful marble statuary which no vandal ever molests. While the government is paternal it is always beneficent and seems to be administered for the greatest good to all alike.

The public parks, and other places of amusement and entertainment, were utilized to make the stay of the dentists and their ladies in the city agreeable. The first evening the Lord Mayor of the city gave a most elaborate reception to the visitors, and every evening some entertainment was provided, the ladies were also beautifully entertained in numerous excursions to interesting places and social functions of various kinds. The Congress was a success and the

Central German Verein has done much to stimulate a new interest of professional advancement. When one gets such a glimpse of dentistry as it is looked upon by all the world it gives him a larger conception of his calling and and makes him wonder what the next 50 or 100 years will do for the health of the people. We cannot close this commentary without noticing the one unfortunate and regretable incident which lost for the Americans much of their cordial endorsement of the management. There seems to be a good bit of feeling between the German Zahnarzt and the Native American dentists practicing in Europe and this also includes the native Germans who have come to America for their professional training. When the Congress was organized no Americans nor Germans who had gotten their degrees in America were invited to assist in organizing the Congress or invited to participate except as associate members. This of course offended all these people, so that very few of them attended the Congress, although eventually the full membership was extended to the Americans. The affair made much hard feeling and we fear it will make still wider the breach between the German Zahnarzt and the American graduates. There should really be no such feeling as there is an abundance of opportunity and crying need for all the well qualified dentists that the German Empire will receive both from its schools at home and abroad for many years to come. Germany is, however, better supplied with dentists than any other European country but it will be a long time before her people have adequate dentistry. It seems to us that there should be no occasion for petty jealousies in so great a cause and with high-minded people.

**UNWISE LEGISLATION.** In another place we print a protest against an evident but probably unintentional restriction of the usefulness of the dentists, because of the enactment of stringent laws regulating the traffic in intoxicating liquors. We do not sympathize with the spirit of this protest of Dr. Zederbaum, nor do we submit graciously to the interpretation of the law by the Michigan State's Attorney General. No sane person can believe that it was the intention of the law makers to prevent dentists from using such remedies as are necessary in the treatment of their patients, and no conviction would ever hold a legally qualified dentist who could show that his use of an alcoholic drug was scientifically indicated and professionally used in the treatment of any disease. It is perfectly proper that dentists as well as physicians should be prohibited from aiding in any way the traffic in spirituous liquors, and we fail to see that any particular stigma has been placed upon the dignity of our profession by this law. The opportunities for prescribing liquors for our patients are so few that we need borrow little trouble because we fear the strong arm of the law. Some malicious person might possibly use this law to one's injury, but we feel perfectly assured that any legitimate use of alcoholic dental remedies will be upheld by a right construction of the law. The cause calling for the enactment of this law is more to be considered than any probable inconvenience that may come to the dental practitioner in the necessary use of his alcoholic remedies.

